# Reply on the comments of Ulla Vogel and colleagues on their recently published paper entitled “Re-evaluation of the occupational exposure limit for ZnO is warranted. Comments on ‘Systemic inflammatory effects of zinc oxide particles: is a re-evaluation of exposure limits needed?’ by Christian Monsé et al.”

**DOI:** 10.1007/s00204-023-03668-0

**Published:** 2024-01-10

**Authors:** Christian Monsé, Rolf Merget, Dirk Pallapies, Thomas Brüning, Jürgen Bünger

**Affiliations:** Institute for Prevention and Occupational Medicine of the German Social Accident Insurance, Institute of the Ruhr-Universität Bochum (IPA), Bürkle-de-La-Camp-Platz 1, 44789 Bochum, Germany

We read with interest the comments (Vogel et al. 2023) regarding the arguments to justify a higher OEL for zinc oxide (ZnO), and hereby, we would like to provide clarifications from a toxicological perspective.

In general, the authors emphasize that chronic acute phase protein elevations, such as C-reactive protein (CRP) and serum amyloid A (SAA), increase the risk of cardiovascular disease (CVD) and that even small elevations are relevant. We disagree. CRP and SAA are useful biomarkers of inflammation, but evidence on their causal role in CVD is still lacking—despite strong scientific effort and hundreds of studies.

A recent review casts doubt that there is a causal link between CRP elevations and CVD at all. Liu and Li (2023) conclude: “Our examination of available studies suggests that CRP is unlikely to be a cause of CVDs. The widely observed associations between CRP and CVDs are more likely to be explained by confounding in observational studies and by treatments in clinical trials.”

The same is true for SAA. In a comprehensive actual review of den Hartigh and coworkers argue: “Elevations of SAA subtypes have been consistently associated with metabolic diseases such as obesity, diabetes, CVD, and autoimmune conditions in humans and in animal models. After 40 years of investigation, evidence is not yet sufficient to determine whether SAA plays causal roles in metabolic disease development and progression, or is merely a biomarker of broader phenomena akin to CRP.” (den Hartigh et al. 2023).

Zinc-specific mechanism of action: We did not define the causal mechanism of action clearly. In our considerations, we focused on the comparison of chemically inert particles with zinc oxide particles (see BaSO_4_ study, Monsé et al. 2022) and attributed the observable systemic inflammation to zinc ions. We agree that other metal oxides can also cause similar effects. Therefore, the term “zinc-specific mechanism of action” should be understood as “metal-specific mechanism of action”.

The authors cite studies showing that insoluble particles should trigger acute phase protein responses. In an experimental human study, Wyatt and coworkers (2020) exposed 20 volunteers to particles (PM 2.5, 37.8 µg/m^3^) collected from the outdoor environment and observed a small increase of SAA. The problem is that the composition of the particle load was not further investigated. Environmental particles are normally very complex mixtures of a broad variety of components containing soluble and insoluble substances. Therefore, the observations cannot be assigned to a substance-specific mechanism (here: mechanism of action of insoluble particles). However, for a singular chemically inert substance such as barium sulfate, we clearly demonstrated that short-term exposure of humans to much higher concentrations (4000 µg/m^3^) did not induce any systemic inflammation.

The cited study by Zhang et al. (2017) has the same disadvantage. Correlations of elevated CRP with PM 2.5 concentrations were investigated and can therefore not be attributed to a specific insoluble component or insoluble components at all.

In the last section of the text, the MAK recommendation was misrepresented. This is 0.1 mg/m^3^ for zinc and not ZnO. The Danish proposal for the occupational exposure limit is given in the text as 0.05 mg/m^3^ ZnO. This presentation could be confusing and should be given as 0.04 mg/m^3^ Zn. The authors conclude that the MAK proposal and the Danish proposal are very similar. Compliance with the concentration level proposed by Vogel et al. would lead to very serious technical problems for the companies concerned and would virtually constitute a ban on work. Even the MAK limit value proposal of 0.1 µg/m^3^ Zn cannot yet be sufficiently complied with by many companies (see Poppe et al. 2019). We, therefore, see the difference between the two proposals as relevant.
